# A Rare CD4−CD8+ Adult T-Cell Leukemia/Lymphoma with Unique Molecular Mutations: A Case Report with Literature Review

**DOI:** 10.1155/2020/8890502

**Published:** 2020-10-30

**Authors:** Jui Choudhuri, Leah Geiser Roberts, Yan Zhang, Yanhua Wang, Yanan Fang

**Affiliations:** ^1^Department of Pathology, Montefiore Medical Center, Bronx, New York City 10467, NY, USA; ^2^Albert Einstein College of Medicine, Pelham PKWY South Bronx, New York City 10467, NY, USA; ^3^Liberty University College of Osteopathic Medicine (LUCOM), Lynchburg, VA, USA; ^4^Jacobi Medical Center and NCB Hospital, Bronx, New York City 10467, NY, USA

## Abstract

Adult T-cell leukemia/lymphoma (ATLL) is a mature T-cell neoplasm caused by infection of the human T-cell lymphotropic virus type 1 (HTLV-1). Most ATLL cases are CD4-positive and CD8-negative. Though rare, there are a few dual negative (CD4−CD8−), dual positive (CD4+CD8+), and CD4−CD8+ cases reported in literature. ATLL is associated with HTLV-1 infection, but HTLV-1 alone cannot cause the malignant transformation of infected T cells. Additional genetic and/or epigenetic events are required for the development of the disease. Here, we report an unusual CD4−CD8+ATLL in a 76-year-old male with a unique molecular genetic profile. Molecular studies revealed alterations in 10 genes. Three of them are predicted to be pathogenic by the computational models, including the frameshift change in ZFHX4 and missense mutations in RHOA and POT1. The specific mutations of POT1 (c.281A > G; p.Q94R), RHOA (c.47G > A; p.C16Y), and ZFHX4 (c.2871delC; p.F958Sfs^*∗*^31) have never been previously reported in ATLL to the best of our knowledge. The clinical significance of other genetic alterations is unknown. Further research is warranted to correlate this patient's molecular findings with other ATLL cases. Correlation specifically with other cases of CD8+ ATLL could prove to be useful in understanding the pathogenesis of this rare variant of an already rare form of leukemia/lymphoma.

## 1. Introduction

Adult T-cell leukemia/lymphoma (ATLL) is a rare and aggressive T-cell neoplasm associated with the human T-cell lymphotropic virus type 1 (HTLV-1) infection [1]. Most ATLL cases involve proliferation of CD4+CD25+ T cells and have been strongly associated with HTLV-1, which preferentially infects CD4+ T cells and leads to clonal expansion. These are typically mature T cells, expressing CD2 and CD5, often negative/dim for CD7, with downregulation of CD3 and T-cell receptor- (TCR-) beta [1]. While ATLL is characteristically the expansion of CD4+CD8−CD25+ T cells, there are occasional cases reported in literature of dual negative (CD4−CD8−), dual positive (CD4+CD8+), and CD4−CD8+ ATLL [2–5]. These unusual ATLL cases have been reported to be more aggressive and with a worse prognosis [2–4].

ATLL is a rare entity in Europe and North America, and it is much more common in populations with endemic HTLV-1 infection such as in southern Japan, sub-Saharan Africa, the Caribbean Basin, and South America [1]. In nonendemic regions, infection and subsequent ATLL can be found most commonly in areas high in immigrant populations. In United States, the incidence of ATLL is approximately 0.05 per 100,000 [6]. The virus can spread via vertical transmission, sexual intercourse, transfusion of contaminated cellular blood products, and use of needles contaminated with the virus (e.g., sharing needles in IVDU) [1]. The mean age of diagnosis of ATLL is approximately 60 years and with no sexual predominance [6]. Familial clustering has been seen, suggesting a possible component of genetic predisposition. Patients can have varied clinical presentation of the disease. The disease is typically subdivided into four clinical presentations: smoldering (indolent), chronic, lymphoma, and acute form [7]. Lymphoma and acute forms are associated with worst prognosis [1].

Studies have been performed trying to find distinct molecular genetic profiles that would predispose a carrier to develop ATLL, but discrete and consistent mutations have not been fully established, and there is still a wide spectrum of mutations being studied. The most common genetic mutations found in ATLL are p16 (30–70% cases) and p53 (10–50% cases) although they are nonspecific [8]. Studies have shown that chromosomal abnormalities are frequent and increase with aggressiveness of the disease. Recent molecular studies have shed a light on the molecular basis of the leukemogenesis of ATLL [9, 10]. We present a rare case of ATLL with a CD4−CD8+ phenotype and the molecular findings associated with it.

## 2. Case Presentation

The case is a 76-year-old male who had moved to the United States from Jamaica, one of the Caribbean islands, five years prior to presenting at a medical facility for unintentional weight loss and night sweats over 3 months and was found to have generalized lymphadenopathy. The patient reported no history of trauma, recent travel, fever, chills, nausea, or vomiting. Past medical history was significant for type 2 diabetes mellitus, hypertension, and remote history of prostate cancer. The initial blood profile showed anemia with a hemoglobin level of 6.7 g/dL and mild thrombocytopenia with a platelet count of 125 × 10^9^/L. White blood cell count was 9.0 × 10^3^/*μ*L with differential count as follows: neutrophils, 89%; lymphocytes, 7%; and monocytes, 3%. Lactate dehydrogenase (LDH) was mildly elevated, and calcium level was within the reference range. A computed tomography (CT) showed diffuse hilar and inguinal lymphadenopathy. Flow cytometry of peripheral blood was negative for involvement by non-Hodgkin B- or T-cell lymphoma. An initial fine needle aspiration cytology was paucicellular with some large atypical cells concerning for lymphoma.

**Figure 1:**
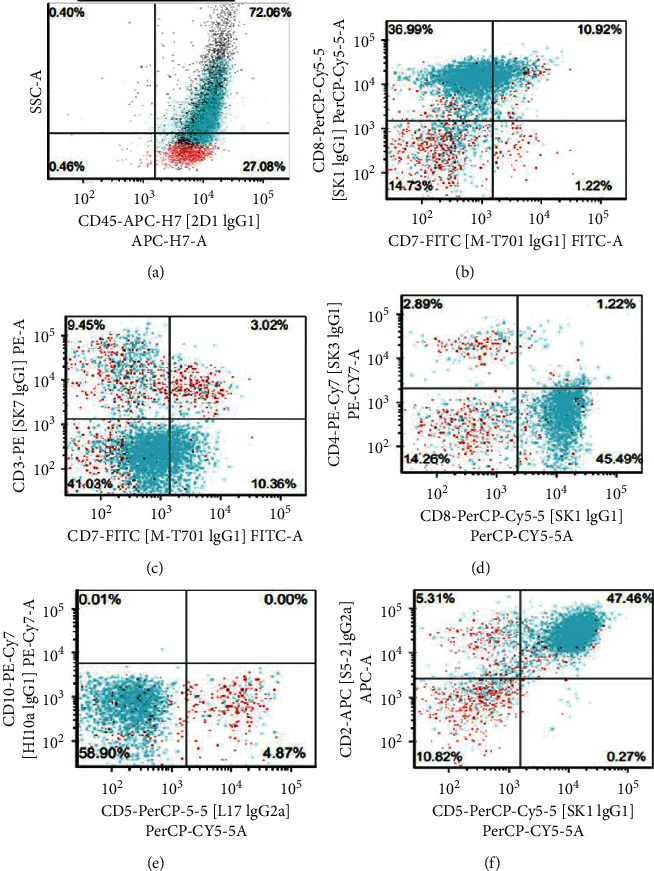
Flow cytometry of the lymph node showing atypical T cells positive for CD2, CD7 (dim), and CD8, with negative surface CD3, CD4, and CD5.

**Figure 2:**
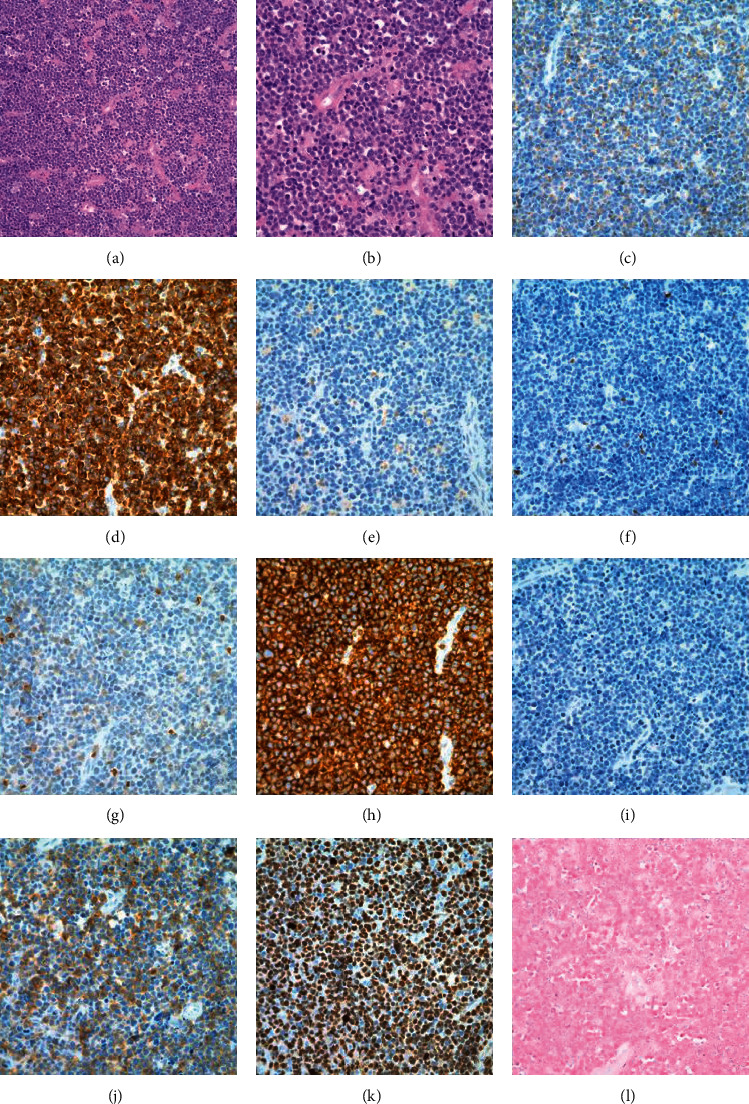
Biopsy of the left cervical lymph node. Hematoxylin and eosin (a, b), immunohistochemistry staining (c–j), and in situ hybridization (l). (a, b) H&E sections showing sheets of pleomorphic atypical medium-to-large lymphocytes. (c) CD2. (d) CD3. (e) CD4. (f) CD5. (g) CD7. (h) CD8. (i) CD20. (j) CD25. (k) Ki67. (l) Epstein–Barr virus- (EBV-) encoded RNAs (EBER) (original magnification, ×200 (a, c–l) and ×400 (b)).

A biopsy was performed on a left cervical lymph node. Flow cytometry showed a population of atypical T cells which were positive for CD2, CD7 (dim), and CD8, while negative for surface CD3, CD5, and CD4 (reference commercial laboratory 1, Figure 1). The hematoxylin and eosin staining of the lymph node revealed complete effacement of the lymph node architecture and replaced by sheets of pleomorphic atypical medium-to-large lymphocytes (Figure 2). The atypical lymphocytes exhibited irregular nuclear contour, condensed chromatin, and distinct nucleoli. By immunohistochemical staining (Figure 2), the atypical lymphocytes were positive for CD3, CD8, and CD25; variably positive for CD2, CD7, and TIA-1; and negative for CD4, CD5, CD20, CD34, TdT, EBER, and CD30. Ki67 demonstrated a high proliferation index (>90%).

Given the suspicion for ATLL, HTLV-1 serology was performed and found to be reactive. HTLV-1 viral sequence was detected in the lymphoma by polymerase chain reaction (PCR) and fluorogenic probe specific for HTLV-1 pol (reference laboratory 2) consistent with the diagnosis of ATLL. Clonal pattern of T-cell receptor gene rearrangement was also detected. Bone marrow biopsy was negative for lymphoma involvement. The patient received six cycles of cyclophosphamide, doxorubicin hydrochloride, Oncovin, and prednisone (CHOP) and intrathecal methotrexate resulting in apparent remission. Six months later, the patient presented with enlarged lymph nodes/facial swelling, and imaging/biopsy result confirmed relapse. The patient received salvage ifosfamide, carboplatin, and etoposide (ICE) therapy. However, there was worsening disease evident on the CT scan, and the patient eventually expired.

Further genetic testing (outside reference laboratory 3) on the lymph node biopsy had revealed ten genomic alterations: DIS3 (c.387-6A > G), ESR1 (c.148G > A; p.A50T), PIK3R1 (c.1205T > C; p.V402A), MYD88 (c.43G > A; p.A15T), PLCG1 (c.1021G > A; p.G341R), POT1 (c.281A > G; p.Q94R), PTEN (c.851A > G; p.E284G), RHOA (c.47G > A; p.C16Y), SAMHD1 (c.184C > T; p.P62S), and ZFHX4 (c.2871delC; p.F958Sfs^*∗*^31). Three mutations identified in DIS3, ESR1, and PIK3R1 were most likely germline alterations given that they were also identified in the peripheral blood sample as well which was not involved by ATLL (on flow cytometry), and the allele frequency was approximately 50%. Three mutations identified in our patient were predicted to be pathogenic including RHOA, ZFHX4, and POT1 genes. The remaining genetic alterations in this case have neither been reported before nor been predicted to be pathogenic using computational models (computational model used by the reference company and/or https://cancer.sanger.ac.uk/cosmic). Thus, those genetic alterations remain of unclear clinical significance at this point.

## 3. Discussion

We report an unusual case of CD4−CD8+ ATLL with unique genetic alterations. Rare cases of CD4−CD8+ have been reported previously [3, 5, 11]. In the study of 107 ATLL cases, Kamihira et al. found the incidence of the CD4−CD8+ to be 4%, while 81% for conventional phenotype CD4+CD8− and 7% each for the dual positive (CD4+CD8+) and dual negative (CD4-CD8-) cases [5]. It is not yet clear whether these CD8+ ATLL cases are due to the infection and transformation of CD8+ T cells directly or modified expression of CD8+ on the CD4+ T cells [12].

ATLL is a mature T-cell neoplasm closely associated with the HTLV-1 infection. The HTLV-1 genome includes the *Gag*, *Pol*, and *Env* genes, and it also includes several regulatory proteins such as Tax and the helix basic-loop zipper protein (HBZ). Tax is known to play a crucial role in initial transformation of cells but is not considered to be a necessary protein for ongoing transformation and progression of established ATLL as it is often found to be absent later down the disease course [13]. While the HBZ RNA promotes proliferation of ATLL cells and plays an important role in the later stages of infection and maintenance of ATLL [10], it has been noted that the onset of ATLL generally occurs following a latency period of approximately 30–50 years after initial HTLV-1 infection, suggesting HTLV-1 viral factors alone are not sufficient for malignant transformation of infected T cells. Other than HTLV-1 viral factors and proteins, additional genetic and/or epigenetic events play a major role in the development of disease. Environmental factors have also been implicated given varying mean age of onset based on geographic location as well as familial clustering [14].

Studies have shown that the mutation rate for ATLL is higher as compared to other malignancies with an average of 2.3 mutations per mega base in coding regions [10]. In ATLL pathogenesis, additional genetic mutations are needed for tumorigenesis other than viral products. Some of the genes involved include p53, CCR4, NOTCH1, and CDKN2A [9, 15]. Recent large-scale integrated molecular studies have identified a number of genes and signaling pathways involved in ATLL [9]. In particular, many genetic alterations were found affecting the TCR/NF-*κβ* pathway and other T-cell-related pathways. For example, the most commonly identified activating mutations include PLCG1, PRKCB, CARD11, VAV1, IRF4, and FYN. Although some involved genes in our case have been reported to be mutated in ATLL or other tumors previously, these specific point mutations and frameshift change have not been reported in ATLL before. Among all the genetic alterations, three of them are predicted to be pathogenic: RHOA, ZFHX4, and POT1.

RHOA is a key regulator in T-cell receptor-/NF-kB signaling pathway and contributes to cytoskeletal regulation, cellular proliferation, and oncogenic transformation. RHOA mutations have been found in ATLL, angioimmunoblastic T-cell lymphoma (AITL), and peripheral T-cell lymphoma with T-follicular helper phenotype, as well as other solid tumors. The mutations in ATLL appear to have unique distribution compared to other T-cell lymphomas and solid tumors [16]. RHOA mutations associated with ATLL are mostly focused around the GTP-binding pocket with greater variance in the discrete mutational hotspots (Cys16, Gly17, and Ala161) [16]. The mutation can be either loss of function or gain of function although both are thought to be involved in ATLL leukemogenesis [16]. By analyzing 12 cases, Nagata et al. also found that different RHOA mutations appear to be associated with distinct immunophenotype and T-cell subsets [16]. The patient in this case did have a missense mutation involving the cysteine at the 16th location, which is one of the above mentioned discrete mutational hotspots associated with ATLL. The patient does not, however, present with the most common mutation (Cys16Arg) [16]. The mutation (c.47G > A; p.C16Y) identified in our patient was reported in a case of acute myeloid leukemia and predicted to be pathogenic based on computational models [17]. The unique mutation may be associated with his rare CD4−CD8+ immunophenotype.

ZFHX4, a member of nucleosome remodeling and deacetylase (NuRD) family, is involved in DNA damage-repair pathway [18]. ZFHX4 mutations have previously been reported in various cancers including multiple myeloma, glioblastoma, and esophageal cancer but not in ATLL. ZFHX4 mutations have been found associated with poorer prognosis in various cancers [18–20]. The molecular pathogenesis of ZFHX4 is not well understood. Limited study showed that knockdown of the ZFHX4 gene led to decreased migration and invasiveness in esophageal squamous cancer cells in vitro [19]. The frameshift mutation of ZFHX4 (c.2871delC; p.F958Sfs^*∗*^31) in this case has never been reported but is expected to be pathogenic.

POT1 mutations are associated with a variety of different malignancies as well [21, 22]. POT1 binds and protects telomeres; loss of this makes the host genome vulnerable to degradation and fusion which ultimately leaves the genome unstable. Mutations in POT1 induce genomic instability through dysfunctional telomeres [21]. Genomic instability puts the cell at risk for genetic alterations that can lead to malignant transformation. The mutation identified (c.281A > G; p.Q94R) has been reported in chronic lymphocytic leukemia but has not been reported in ATLL yet [21, 23]. This mutation is also predicted to be pathogenic by the computational model [24].

Among other genetic alterations identified in this case, three of them (DIS3, ESR1, and PIK3R1) are most likely germline alterations. The remaining four genetic alterations in PTEN, MYD88, PLCG1, and SAMHD1 have never been reported, and the clinical significance is unknown, which does not exclude the possible contribution to the disease. Among these genes, PLCG1 is a key molecule involved in T-cell receptor- (TCR-) nuclear factor- (NF-) *κ*B signaling, and its activating mutations have been commonly found in ATLL cases (36%) [9]. PTEN-mediated PI3K/AKT pathway activation is also involved in ATLL leukemogenesis [25]. MYD88 plays an important role in the pathogenesis of B-cell lymphomas and other tumors [26]. However, the missense mutations detected in this case including PLCG1 (c.1021G > A; p.G341R), PTEN (c.851A > G; p.E284G), and MYD88 (c.43G > A; p.A15T) have neither been reported in any tumor nor been predicted to be pathogenic by the computational models.

## 4. Conclusion

Given the current available data, RHOA, POT1, and ZFHX4 are the most likely culprits involved in this patient's leukemogenesis, based on the various databases and literature reviews. However, the significance of the others is not excluded. It is possible that other identified mutations are involved in the progression of this patient's HTLV-1 infection to CD4−CD8+ ATLL. More investigation and research are warranted for specially studying ATLL in different geographical regions. However, it is challenging given the extremely small sample size of unusual phenotypic cases such as ours, CD4−CD8+ ATLL. The overall prognosis of ATLL continues to be poor and with a high relapse rate despite aggressive chemotherapy. Further advances in the molecular aspects might be a clue to the therapeutic course for this disease.
